# Blood pressure variability and its association with echocardiographic parameters in hypertensive diabetic patients

**DOI:** 10.1186/s12872-015-0183-1

**Published:** 2016-01-08

**Authors:** Daniela Massierer, Liana Farias Leiria, Mateus Dorneles Severo, Priscila Dos Santos Ledur, Alexandre Dalpiaz Becker, Fernanda Mus Aguiar, Eliandra Lima, Valéria Centeno Freitas, Beatriz D. Schaan, Miguel Gus

**Affiliations:** Cardiology, Hospital de Clínicas de Porto Alegre, Rua Ramiro Barcelos 2350, sala 2061, 90035-003 Porto Alegre, RS Brazil; Endocrine Division, Hospital de Clínicas de Porto Alegre, Rua Ramiro Barcelos 2350, sala 2061, 90035-003 Porto Alegre, RS Brazil; Postgraduate Studies Program in Cardiology, School of Medicine, Universidade Federal do Rio Grande do Sul, Rua Ramiro Barcelos, 2400 - 2° andar, Porto Alegre, RS Brazil; Faculty of Medicine, Universidade Federal do Rio Grande do Sul, Rua Ramiro Barcelos, 2400 Porto Alegre, RS Brazil; Universidade Federal de Ciências da Saúde de Porto Alegre, Rua Sarmento Leite, 245, 90050-170 Porto Alegre, RS Brazil

**Keywords:** Diabetes mellitus type 2, Echocardiography, Hypertension

## Abstract

**Background:**

Blood pressure (BP) variability is associated with target organ damage in hypertension and diabetes. The 24 h ambulatory blood pressure monitoring (24 h-ABPM) has been proposed as an evaluation for BP variability using several indexes [standard deviation (SD) of mean BP, coefficient of variation (CV), BP variation over time (time-rate index)].

**Methods:**

We evaluated the association between BP variability measured by 24 h-ABPM indexes and echocardiographic variables in a cross-sectional study in 305 diabetic-hypertensive patients.

**Results:**

Two groups were defined by the median (0.55 mmHg/min) of time-rate systolic BP (SBP) index and classified as low or high variability. Age was 57.3 ± 6.2 years, 196 (64.3 %) were female. Diabetes duration was 10.0 (5.0–16.2) years, HbA1c was 8.2 ± 1.9 %. Baseline clinical characteristics were similar between low (*n* = 148) and high (*n* = 157) variability groups. Office SBP and systolic 24 h-ABPM were higher in the high variability group (139.9 mmHg *vs* 146.0 mmHg, *P* = 0.006; 128.3 mmHg *vs* 132.9 mmHg, *P* = 0.019, respectively). Time-rate index, SD and CV of SBP, were higher in high variability group (*P* < 0.001; *P* < 0.001 and *P* = 0.003, respectively). Time-rate index was not independently associated with the echocardiography’s variables in multiple linear model when adjusting for age, 24 h-ABPM, diabetes duration and HbA1c. The multiple linear regression model revealed that the significant and independent determinants for septum thickness, relative wall thickness and posterior wall thickness (parameters of left ventricular hypertrophy) were: age (*p* = 0.025; *p* = 0.010; *p* = 0.032, respectively) and 24 h-SBP (*p* < 0.001 in the three parameters).

**Conclusion:**

BP variability estimated by 24 h-ABPM is not independently associated with echocardiographic parameters in diabetic-hypertensive patients.

## Background

Observational studies had consistently shown the continuous relationship between office systolic and diastolic blood pressure (BP) and cardiovascular events [1]. Indeed, the causal role of high BP for cardiovascular disease was fully confirmed by clinical trials [2]. The evidence that high BP highers the risk for cardiovascular events, and the consistent reduction of these events by clinical trials of BP-lowering agents are robust proofs of the concept that high BP is a major cardiovascular determinant [3].

Methods of out-of-office BP measurement such as 24 h ambulatory BP monitoring (24 h-ABPM) evaluated in general population or in hypertensive-based longitudinal studies also showed a close relationship between BP elevation and cardiovascular risk [4, 5]. Other parameters assessed by 24 h-ABPM, beyond the average of BP, such as BP variability, may provide additional information regarding the cardiovascular risk [6, 7]. Blood pressure fluctuations are a result of the interplay between external environmental stimuli, vascular environment and biological autonomic circulatory regulation [8]. Measures of BP variability can be obtained through different methods or indexes [9] and short-term BP variability over a 24 h period estimated from 24 h-ABPM can be measured by a more refined estimation such as the time-rate index. This index is calculated as the mean of the absolute ratios of the differences between successive BP measures and the time (in minutes) between them. It quantifies how fast and in which direction systolic BP (SBP) values change and, thereby, is claimed to offer an insight into how steep these changes are. Cross-sectional and longitudinal studies showed an independent relationship between the time-rate index and target-organ damage or cerebrovascular events [10–13]. Echocardiographic evaluation is recommended to assess asymptomatic organ damage in hypertensive patients since left ventricular hypertrophy and diastolic dysfunction are independently associated with cardiovascular outcomes [14]. These variables could be used as surrogates to assess the possible association between BP variability and cardiovascular risk in high-risk patients.

Considering the high cardiovascular risk profile [15] and the frequent occurrence of autonomic dysfunction in diabetic patients, [16] the relationship between the short-term variability over a 24 h period and target-organ damage should be estimated. This relationship has not been well evaluated in observational studies [17]. The present cross-sectional study aims to address this issue by evaluating the potential association between variables of BP variability including the time-rate index of 24 h-SBP and echocardiographic parameters of cardiac chambers, left ventricular hypertrophy and diastolic function.

## Methods

This is a cross-sectional study conducted in the outpatient clinic of a tertiary hospital (Hospital de Clínicas de Porto Alegre/Brazil), from April 2010 to December 2011. The data came from a larger study that aimed to assess cardiovascular risk in diabetic hypertensive patients through non-invasive methods [18–20]. The study was approved by the Ethics Committee of Porto Alegre Clinics Hospital (Hospital das Clínicas de Porto Alegre- GPPG 09–636) which is accredited by the Office of Human Research Protections as an Institutional Review Board. All participants signed an informed consent form before entering the study.

**Fig. 1:**
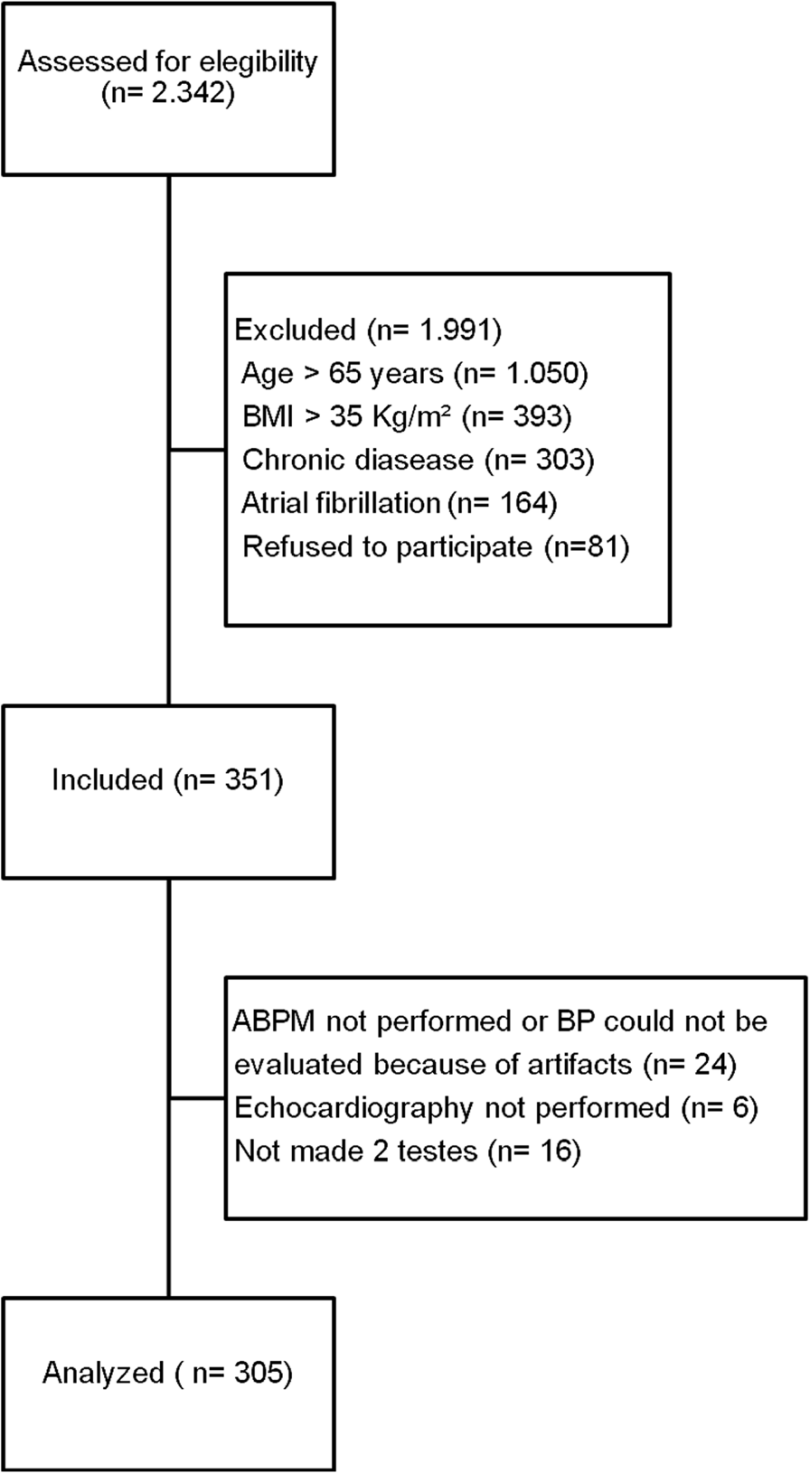
Flowchart of patient’s selection

The study population was selected from a consecutive sample of 2342 screened patients. Patients were included in this analysis if they had a previous diagnosis of type 2 diabetes mellitus and hypertension, as ascertained by their personal history of the diseases or because they were using antidiabetic and/or antihypertensives for treatment, and were less than 65 years of age. Exclusion criteria were body mass index (BMI) ≥ 35 kg/m,^2^ cancer, arrhythmias (e.g., atrial fibrillation) that could interfere with BP measurement and 24 h-ABPM recordings. According to these criteria, 351 patients were included (Fig. 1).

Patients who met the inclusion criteria and agreed to participate underwent a demographic and clinical baseline data collection, including the assessment of duration of diabetes and hypertension and its known chronic complications, smoking habits, previous cardiovascular diseases, medication in use, BMI, and office BP levels.

Blood pressure was measured after 15 min of rest with an automatic sphygmomanometer (OMRON Comfort III Visomat Incoterm, Germany). High office BP levels were defined as office BP higher than 140/90 mmHg.

Among the 351 selected individuals, 93.1 % (*n* = 327) underwent 24 h-ABPM (Spacelabs 90207, Redmond, WA) on an usual working day, performed at up to four months after the initial evaluation (approximately 75 % of patients had full evaluation within 30 days). Readings were obtained automatically at 15-min intervals during the day and at 20-min intervals during the night for the duration of the 24 h-ABPM period. Cuff size was chosen according to arm circumference. Daytime was defined as the interval between 06:00–22:00 h and nighttime was the interval between 22:00–06:00 h. Individuals with less than 6 and 18 measures during the night and the day periods, respectively (*n* = 24) were excluded from further analysis. All individuals were instructed to rest or sleep during the nighttime and to maintain their usual activities during daytime. Based on the results of the 24 h-ABPM, the mean 24 h-SBP and diastolic BP (DBP) were calculated for each patient. We calculated three different parameters of SBP variability: the standard deviation of mean (SD), coefficient of variation (CV = SD/mean pressure × 100%) and rate of change in SBP over time (mmHg/min), defined as the first derivative values of SBP by time (time-rate index). This index allows the calculation of the sum of angular coefficients and aims to measure how fast or how slow and which direction SBP values change. The measure was calculated using the following formula [9]:

$$ R=\left|\overline{r}\right|=\frac{{\displaystyle {\sum}_{i=1}^{N-1}\left|{r}_i\right|}}{N-1} $$

In the formula, *r* is the rate of BP variability over time (considering the differences between BP measurements in each time interval) and *N* is the number of recordings.

Echocardiography was performed in 98.3 % (*n* = 345) of patients by a single investigator, usually on the same day of the 24 h-ABPM. Images were obtained using a commercially available instrument (GE Healthcare VIVID 7, Buckinghamshire, UK) equipped with a 4 MHz transducer, according to the recommendations of the American Society of Echocardiography, [21] using three consecutive cardiac cycles. Standard parasternal and apical views were performed with subjects in the partial left decubitus position. Left ventricular volumes and ejection fraction were calculated by the Simpson's formula; ventricular mass was calculated based on wall thickness adjusted in two ways: to the body surface area and indexed to body height to the power of 2.7. Relative wall thickness (RWT) was defined as “septum + posterior wall (PW) thickness” divided by “left ventricular diastolic diameter”. Diastolic function was evaluated based upon mitral inflow doppler measurements (maximum early flow velocity in diastole- E wave- and maximum late velocity flow in diastole- A wave). Peak early (E’) and peak late (A’) tissue Doppler velocities were assessed at the mitral annulus, determining values as the average of septal and lateral wall measurements. The variables septum and PW thickness, RWT and left ventricular mass índex were used for categorical analyzes on the prevalence of left ventricular hypertrophy, adopting the reference values proposed by the American Society of Echocardiography [21]. Hypertrophy was defined considering the normal range of 1.0 cm to septum obtained in a representative sample of adults in the city of Porto Alegre, as previously described [22].

Fasting blood samples were collected for laboratory analysis using commercial kits. Plasma glucose was evaluated by a glucose oxidase method, serum creatinine by Jaffé’s reaction, and glycated hemoglobin (HbA1c) by ion-exchange HPLC (Merck-Hitachi L-9100 HbA_1c_ analyzer; reference range 4.8–6.0 %; Merck, Darmstadt, Germany). Serum total cholesterol and triglycerides were measured by enzymatic-colorimetric methods (Merck Diagnostica, Germany; Boehringer Mannheim, Argentina), and High-density lipoprotein (HDL) cholesterol by a homogeneous direct method (autoanalyzer, ADVIA 1650). Low-density lipoprotein (LDL) cholesterol was calculated using Friedewald’s formula [23]. Glomerular filtration rate was calculated using the MDRD (Modification of diet in renal disease) equation [24]. C-Reactive Protein was measured using an ultrasensitive assay by nephelometry (Bayer nephelometer, Leverkusen, Germany), capable of evaluating values in the range of 1–4 mg/l. Urinary albumin excretion was evaluated by immunoturbidimetry (MICROALB- AMES Kit, CA, USA). Abnormal albuminuria was defined as albuminuria of 17 mg/dl or more [25].

### Statistical analyses

The comparison groups were defined by the median of time-rate index of 24 h-SBP and classified as low and high variability of time-rate index: values ≤ 0.54 mmHg/min or ≥ 0.55 mmHg/min, respectively. Comparisons were tested by Pearson’s chi-square test, Student’s *t* test, and Mann–Whitney test. Logistic regression models and multiple linear regression were used to evaluate the association between echocardiography’s variables and parameters of variability of 24 h-SBP. Age, 24 h-SBP, diabetes duration (years) and HbA1c were included in models. Continuous variables are expressed as mean ± SD or median and interquartile range. Categorical variables are expressed as number (%).

Sample size calculation was based upon the mean differences in two echocardiographic variables of septum and E/E’, between low and high variability. Considering the 1:1 proportion in low and high variability groups, a SD of 0.17 cm, an alpha error of 5 %, and power of 90 % to detect a 10 % increase in the septum, the sample size estimation was 124 patients (62 in each group). For the E/E’ ratio we considered a SD of 3.6, considering the same proportion, alpha error and power to detect a 15 % of difference between groups, the sample size estimation was 162 patients (81 in each group).

Logarithmic transformation was applied to albuminuria before parametric tests were applied. *P* values < 0.05 (two-tailed) were considered to be statistically significant. Statistical Package for Social Science (SPSS, Chicago, IL.) version 18.0 was used for the analyses.

## Results

A total of 305 patients was evaluated. The characteristics of the subjects studied, grouped as low and high variability (time-rate index of 24 h-SBP) are presented in Table 1. Patients were 57.3 ± 6.2 years, 196 (64.3 %) were women, and 207 (68.3 %) were caucasian. Body mass index, previous history of stroke, use of statins were higher in low variability group; insulin use was higher in the high variability group. Previous history of any cardiovascular disease was present in 88 patients (29.4 %), but was similar between groups. The other characteristics were similar in both groups.

**Table 1:** Clinical characteristics of the participants according to blood pressure variability

Characteristics	Total sample	Low variability	High variability	*P*
	(*n* = 305)	(*n* = 148)	(*n* = 157)	
Age	57.3 ± 6.2	57.9 ± 6.4	57.5 ± 6.0	0.565
Female gender	196 (64.3)	99 (66.9)	97 (61.8)	0.354
Caucasian	207 (68.3)	99 (66.9)	110 (70.1)	0.334
Duration of diabetes (years)	10 (5 – 16)	10 (5 – 16)	10 (5 – 17)	0.466
Weight	78.3 ± 12.8	79.6 ± 13.6	77.3 ± 12.1	0.116
BMI (kg/m^2^)	30.1 ± 3.8	30.7 ± 3.9	29.6 ± 3.7	0.013
Waist circumference	102 ± 9	103 ± 10	101 ± 91	0.203
Neck circumference	38.5 ± 3.5	38.5 ± 3.3	38.6 ± 3.7	0.874
Smoking				
No	163 (33.4)	81 (55.9)	82 (52.2)	0.803
Yes	38 (12.6)	18 (12.4)	20 (12.7)	
Former	101 (54)	46 (31.7)	55 (35.0)	
With any previous cardiovascular comorbidity^a^	70 (23)	31 (20.9)	39 (24.8)	0.404
Abnormal albuminuria^b^	88 (28.9)	43 (29.0)	45 (28.7)	0.520
Myocardial infarction	38 (12.6)	16 (11.0)	22 (14.1)	0.410
Coronary artery bypass grafting	13 (4.3)	4 (2.8)	9 (5.8)	0.199
PCI	25 (8.3)	9 (6.3)	16 (10.3)	0.210
Heart failure	26 (8.7)	15 (10.3)	11 (7.1)	0.318
Stroke	28 (9.5)	19 (13.5)	9 (5.8)	0.024
Medications				
Insulin	143 (47.2)	58 (39.5)	85 (54.5)	0.009
1 Antihypertensive drug	50 (16.4)	22 (14.9)	28 (17.8)	0.537
2 Antihypertensive drug	87 (28.5)	38 (25.7)	49 (31.2)	0.311
≥3 Antihypertensive drug	168 (55.1)	88 (59.5)	80 (51)	0.167
Antiplatelet	199 (65.9)	101 (68.7)	98 (63.2)	0.315
Statins	210 (69.8)	113 (76.9)	97 (63)	0.009
Laboratory characteristics				
HbA1c (%)	8.2 ± 1.9	8.2 ± 1.9	8.3 ± 1.9	0.743
Fasting plasma glucose (mg/dL)	159.3 ± 72.4	162.2 ± 74.8	156.7 ± 70.4	0.533
Total cholesterol (mg/dL)	178.8 ± 42.4	176.6 ± 38.7	180.7 ± 45.6	0.426
HDL cholesterol (mg/dL)	41.9 ± 11.8	42.1 ± 12.3	41.8 ± 11.4	0.793
Triglycerides (mg/dL)	155 (103.8 – 234)	152 (103 – 216)	163 (104 – 248)	0.534
GFR (mL/min/1.73 m^2^)	90.4 ± 26.8	89.1 ± 26.4	91.7 ± 27.2	0.401

The comparison groups were defined by the median of time-rate index of 24 h systolic BP and classified as low and high variability of time-rate index: values ≤ 0.54 or ≥ 0.55, respectively

*BMI* body mass index; *PCI* percutaneous coronary intervention; *GFR* estimated glomerular filtration rate calculated by the MDRD equation; BP: blood pressure

^a^With previous cardiovascular comorbidity = when reported at least one previous cardiovascular disease (Myocardial infarction; Coronary artery bypass grafting; PCI; Heart failure; Stroke)

^b^Abnormal albuminuria, defined by albuminuria >17 mg/L

Continuous variables are expressed as mean ± standard deviation or median (interquartile range (p_25_-p_75_)). Categorical variables are expressed as number (%). Comparisons (low variability vs. high variability) were tested by Pearson’s *χ* ^2^ test, Student *t* test and Mann–Whitney test

Office BP recordings and 24 h-ABPM parameters are presented in Table 2. Systolic BP was 6.1 mmHg (*P* = 0.006) higher in the high variability as compared to the low variability group, as well as mean and daytime SBP of 24 h-ABPM (*P* = 0.019 and *P* < 0.001, respectively). The time-rate index of 24 h-SBP was higher in the high variability group as compared to the low variability group (0.648 mmHg/min *vs* 0.459 mmHg/min, respectively; *P* < 0.001), such as the other parameters of variability, SD SBP (13.76 mmHg *vs* 11.37 mmHg, respectively) and CV of SBP (10.99 % *vs* 8.89 %, respectively) were higher in the group of high variability when compared to the group of low variability.

**Table 2:** Office blood pressure recordings and ambulatory blood pressure monitoring parameters of the participants according to blood pressure variability

	Total sample	Low variability	High variability	P
	(*n* = 305)	(*n* = 148)	(*n* = 157)	
Office SBP (mmHg)	143.0 ± 19.3	139.9 ± 17.5	146.0 ± 20.6	0.006
Office DBP (mmHg)	82.0 ± 10.6	81.4 ± 10.6	82.6 ± 10.7	0.355
24 h-ABPM SBP (mmHg)	130.6 ± 16.9	128.3 ± 14.7	132.9 ± 18.6	0.019
24 h-ABPM DBP (mmHg)	76.5 ± 9.3	75.7 ± 10.0	77.3 ± 8.6	0.144
Daytime 24-ABPM SBP (mmHg)	133.7 ± 15.9	130.5 ± 14.5	136.9 ± 16.7	<0.001
Daytime 24-ABPM DBP (mmHg)	78.9 ± 10.0	77.9 ± 10.4	79.9 ± 9.7	0.080
Nighttime 24-ABPM SBP (mmHg)	124.0 ± 18.3	122.6 ± 18.4	125.5 ± 18.2	0.169
Nighttime 24-ABPM DBP (mmHg)	69.6 ± 10.7	69.8 ± 11.2	69.5 ± 10.4	0.817
Time-rate index SBP (mmHg/min)	0.557 ± 0.116	0.459 ± 0.058	0.648 ± 0.075	<0.001
SD SBP (mmHg)	12.60 ± 4.40	11.37 ± 4.24	13.76 ± 4.25	<0.001
CV SBP (%)	9.97 ± 6.29	8.89 ± 3.16	10.99 ± 8.10	0.003

The comparison groups were defined by the median of time-rate index of 24 h systolic BP and classified as low and high variability of time-rate index: values ≤ 0.54 or ≥ 0.55, respectively

*BP* blood pressure, *ABPM* ambulatory blood pressure monitoring, *SBP* systolic blood pressure, *DBP* diastolic blood pressure, *SD SBP* standard deviation of mean SBP, *CV SBP* coefficient of variability SBP

Data are expressed as mean ± standard deviation. Comparisons (low variability vs. high variability) were tested by Student *t* test

Echocardiographic measurements are presented in Table 3. From the total sample, 178 patients (58.4 %) had ventricular hypertrophy considering a cut-off point of 1 cm. When stratified by gender and considering the threshold values for the population of Porto Alegre [22] the proportions were 93.6 % and 95.9 % for men and women, respectively. Considering de diastolic function and the cut-off point >8 for the E/E’ ratio, 234 patients (76.8 %) showed abnormal values [21].

**Table 3:** Echocardiographic parameters of the participants according to blood pressure variability

	Total sample	Low variability	High variability	P
	(*n* = 305)	(*n* = 148)	(*n* = 157)	
Cardiac chamber diameters				
Aorta (cm)	3.16 ± 0.36	3.17 ± 0.37	3.14 ± 0.34	0.383
Left atrium (cm)	3.80 ± 0.45	3.83 ± 0.46	3.77 ± 0.42	0.238
LVSD (cm)	2.99 ± 0.40	2.99 ± 0.42	2.99 ± 0.39	0.898
LVDD (cm)	4.58 ± 0.47	4.61 ± 0.47	4.55 ± 0.46	0.291
Right ventricle (cm)	2.15 ± 0.28	2.16 ± 0.27	2.13 ± 0.28	0.470
LVEF (%)	64.66 ± 5.27	64.90 ± 5.09	64.42 ± 5.44	0.856
Left ventricular hypertrophy				
RWT	0.43 ± 0.08	0.43 ± 0.09	0.43 ± 0.07	0.780
Septum thickness (cm)	1.00 ± 0.17	1.00 ± 0.18	0.99 ± 0.16	0.676
PW Thickness (cm)	0.95 ± 0.15	0.95 ± 0.15	0.95 ± 0.15	0.683
LVMI (g/m^2^)	99.19 ± 30.24	100.38 ± 32.24	98.08 ± 28.33	0.513
LAVI (mL/m^2^)	29.14 ± 9.98	29.32 ± 10.64	28.97 ± 9.34	0.497
Diastolic function				
E wave velocity (m/s)	0.98 ± 0.18	0.97 ± 0.20	0.99 ± 0.16	0.494
A wave velocity (m/s)	0.82 ± 0.23	0.82 ± 0.24	0.82 ± 0.23	0.976
E wave DT (m/s)	235.28 ± 44.16	233.07 ± 45.26	237.37 ± 43.13	0.397
A wave length (cm/s)	179.15 ± 42.30	176.44 ± 42.15	181.71 ± 42.41	0.292
E/A ratio (m)	0.95 ± 0.30	0.94 ± 0.28	0.95 ± 0.33	0.795
E’ wave velocity (m/s)	0.07 ± 0.03	0.07 ± 0.01	0.08 ± 0.04	0.387
E/E’ ratio	11.10 ± 3.66	11.22 ± 4.08	10.99 ± 3.22	0.586
IVRT (m/s)	109.22 ± 18.15	109.42 ± 19.11	109.04 ± 17.24	0.855

The comparison groups were defined by the median of time-rate index of 24 h systolic BP and classified as low and high variability of time-rate index: values ≤ 0.54 or ≥ 0.55, respectively

*LVSD* left ventricular systolic diameter, *LVDD* left ventricular diastolic diameter, *LVEF* left ventricular ejection fraction, *RWT* relative wall thickness, *PW* posterior wall, *LVMI* left ventricular mass index, *LAVI* left atrial volume index, *IVTR* isovolumetric relaxation time, *DT* E wave deceleration time, *BP* blood pressure

Data are expressed as mean ± standard deviation. Comparisons (low vs. high variability) were tested by Student *t* test

The time-rate index of SBP was not associated with the echocardiography’s variables in multiple linear model when adjusting for age, 24 h-SBP, duration of diabetes (years) and HbA1c (Table 4). The multiple linear regression model revealed that the significant and independent determinants for septum thickness, RWT and PW thickness (parameters of left ventricular hypertrophy) were: age (*p* = 0.025; *p* = 0.010; *p* = 0.032, respectively) and 24 h-SBP (*p* < 0.001 in the three parameters). Considering the parameters of diastolic function, age was the only variable that was significantly associated with isovolumetric relaxation time (IVRT) and E/E’ ratio.

**Table 4:** Association between time-rate index and echocardiography’s variables in multiple linear model

	Beta	S.E.	*P*
Left ventricular hypertrophy			
Septum thickness (cm)			
Time-rate SBP (mmHg/min)	−0.029	0.086	0.739
Age (years)	0.004	0.002	0.025
24 h-ABPM SBP (mmHg)	0.002	0.001	<0.001
Diabetes duration (years)	<0.001	0.001	0.510
HbA1c (%)	0.003	0.005	0.635
RWT			
Time-rate index SBP (mmHg/min)	−0.044	0.041	0.277
Age (years)	0.002	0.001	0.010
24 h-ABPM SBP (mmHg)	0.001	<0.001	<0.001
Diabetes duration (years)	0.001	0.001	0.134
HbA1c (%)	0.001	0.003	0.583
PW Thickness			
Time-rate SBP (mmHg/min)	0.006	0.075	0.937
Age (years)	0.003	0.001	0.032
24 h-ABPM SBP (mmHg)	0.002	<0.001	<0.001
Diabetes duration (years)	<0.001	0.001	0.591
HbA1c (%)	<0.001	0.005	0.977
Diastolic function			
IVRT (m/s)			
Time-rate SBP (mmHg/min)	−5.243	9.248	0.571
Age (years)	0.889	0.174	<0.001
24 h-ABPM SBP (mmHg)	0.041	0.054	0.443
Diabetes duration (years)	−0.139	0.130	0.284
HbA1c (%)	0.644	0.581	0.269
E/E’ ratio			
Time-rate SBP (mmHg/min)	0.419	1.911	0.826
Age (years)	0.128	0.036	<0.001
24 h-ABPM SBP (mmHg)	0.004	0.011	0.744
Diabetes duration (years)	0.003	0.027	0.899
HbA1c (%)	−0.042	0.120	0.727

*BP* blood pressure, *SBP* systolic blood pressure, *ABPM* ambulatory blood pressure monitoring, *HbA1c* glycated hemoglobin, *RWT* relative wall thickness, *PW* posterior wall, *IVRT* isovolumetric relaxation time. Adjusted for age, 24 h-ABPM-hour ABPM SBP, duration of diabetes (years) and HbA1c

The other variability parameters were also analyzed, but also showed no significant differences between low and high variability groups.

## Discussion

The results of this cross-sectional study in a sample of hypertensive-diabetic patients did not show associations between BP variability assessed through 24 h-ABPM with echocardiographic variables related to diastolic function, left ventricular hypertrophy and cardiac chamber diameters. The variables significantly associated with parameters of left ventricular hypertrophy (septum thickness, RWT and PW thickness) and diastolic function (IVRT and E/E’) were age and 24 h-SBP, and the only parameter associated with diastolic function (IVRT and E/E’) was age.

Cross-sectional and longitudinal studies showed a positive association between BP variability and cardiovascular risk in pure hypertensive patients. The identification of higher risk patients through variables beyond the absolute values of BP could have practical applications. High risk patients with high BP variability could be chosen to have lower BP targets. Moreover, there is some evidence of a class difference effect between antihypertensive drugs in the within-individual visit-to-visit variability of BP. This potential effect on BP variability could be the guide to an optimal antihypertensive treatment in higher risk patients [26]. In a prospective study, Zis et al. [13] reported that patients with higher 24 h rates of SBP variation assessed by the time-rate index were more likely to have a negative neurologic outcome at 1 year after stroke. Moreover, a cross-sectional study with 539 subjects showed an independent association between time-rate index of 24 h-SBP and intima-media thickness of the carotid measured by ultrasound [9]. However, the authors did not define the cutoff points of BP variability normality. In another cross-sectional study 24 h-BP variability of SBP was independently associated with impaired renal function [12].

The association of time-rate index of SBP with echocardiographic parameters was previously studied by Zakopoulos et al. in a cross-sectional study. They demonstrated that a 0.1 mmHg/min increase in the daytime rate of SBP variation was associated with an increment of 7.087 g (95 % confidence interval 4.775–9.399) in the left ventricular mass [27]. Short-term BP variability can be estimated through different indexes derived from 24 h-ABPM. Mena et. al in a longitudinal study with 312 hypertensive patients identified an independent relationship between the “average real variability index”, an index that also averages the absolute differences of consecutive measurements, and cardiovascular events. This positive relationship was not identified with the SD of the mean SBP [28]. Despite these evidences, guidelines do not recommend the use of BP variability parameters for routine clinical use in hypertensive patients, mainly because the lack of threshold values of BP variability and the absence of evidences of any intervention effect [7, 29].

Diabetes is associated with higher values of short-term BP variability [30]. Ozawa et al. in a prospective study with diabetic hypertensive patients demonstrated higher values of 24 h-SBP and DBP variability than the non-diabetic hypertensive group (SD of mean SBP, 18.2 mmHg *vs* 14.5 mmHg; *p* = 0.041 and SD of mean DBP, 11.5 mmHg *vs* 9.6 mmHg; *p* = 0.042). A prospective study in patients with type 2 diabetes has shown that nighttime BP variability estimated by SD of the nighttime SBP and DBP was an independent predictor of future incidence of cardiovascular events [31]. However in other reports in diabetic patients, especially with nephropathy and sympathovagal imbalance, there was an absence of nighttime BP falling because of functional impairment of the autonomic nervous system [32, 33]. Unfortunately, information on diabetic neuropathy, which could have influenced the BP variability, was not available in our study.

In the present study we analyzed the relationship of short-term BP variability and echocardiographic parameters in a high risk sample. Diabetic hypertensive patients have approximately twice the risk of developing cardiovascular events when compared to purely hypertensive patients. Moreover, 56 % of our sample had history of previous cardiovascular disease, the median duration of diabetes was 10 years, the mean HbA1c was 8.2 ± 1.9 % and almost 60 % had left ventricular hypertrophy. This high-risk profile of the subjects evaluated can explain our negative results, as parameters of BP variability may not add cardiovascular risk information beyond age or BP values in such a high risk sample.

Certain limitations of the present study should be acknowledged. Firstly, we quantified the rate of BP changes using discontinuous 24 h-ABPM techniques, which cannot adequately assess short-lasting BP fluctuations and can only provide some insight into slow and relatively ‘long-term’ BP oscillations. Secondly, the echocardiographic phenotypes assessed are not in fact endpoints. However, in longitudinal studies, these echocardiographic abnormalities have been associated with hard outcomes. Several studies have also confirmed the prognostic significance of left ventricular hypertrophy and diastolic dysfunction [34, 35]. Therefore, the detection and quantification of these echocardiographic variables seems to be relevant in the monitoring of target-organ damage in diabetic hypertensive patients.

## Conclusion

In conclusion, in a diabetic hypertensive high risk sample, BP variability estimated by time-rate índex of SBP was not independently associated with echocardiographic parameters of left ventricular hypertrophy or diastolic function. The use of BP variability for risk stratification beyond the absolute level of BP in this clinical setting should be questioned. Prospective studies in diabetic hypertensive patients with hard outcomes could better confirm our findings.

## Abbreviations

24 h-ABPM: 24 h ambulatory BP monitoring
BMI: body mass index
BP: blood pressure
CV: coefficient of variation
DBP: diastolic BP
HbA1c: glycated hemoglobin
HDL: high-density lipoprotein
IVRT: isovolumetric relaxation time
LDL: low-density lipoprotein
PW: posterior wall
RWT: relative wall thickness
SBP: systolic BP
SD: standard deviation
